# Age-Independent Preoperative Chemosensitivity and 5-Year Outcome Determined by Combined 70- and 80-Gene Signature in a Prospective Trial in Early-Stage Breast Cancer

**DOI:** 10.1245/s10434-022-11666-2

**Published:** 2022-04-04

**Authors:** Pat Whitworth, Peter D. Beitsch, James V. Pellicane, Paul L. Baron, Laura A. Lee, Carrie L. Dul, Charles H. Nash, Mary K. Murray, Paul D. Richards, Mark Gittleman, Raye Budway, Rakhshanda Layeequr Rahman, Pond Kelemen, William C. Dooley, David T. Rock, Ken Cowan, Beth-Ann Lesnikoski, Julie L. Barone, Andrew Y. Ashikari, Beth Dupree, Shiyu Wang, Andrea R. Menicucci, Erin B. Yoder, Christine Finn, Kate Corcoran, Lisa E. Blumencranz, William Audeh, NBRST Investigators Group

**Affiliations:** 1 Nashville Breast Center, Nashville, TN USA; 2 Targeted Medical Education, Cupertino, CA USA; 3 Dallas Surgical Group, Dallas, TX USA; 4 Bon Secours Cancer Institute, Richmond, VA USA; 5 Breast and Melanoma Specialist of Charleston, Charleston, SC USA; 6 Lenox Hill Hospital/Northwell Health, New York, NY USA; 7 Comprehensive Cancer Center, Palm Springs, CA USA; 8 Ascension St. John Hospital Great Lakes Cancer Management Specialists, Grosse Pointe Woods, MI USA; 9 Northeast Georgia Medical Center, Gainesville, GA USA; 10 Akron General Medical Center, Akron, OH USA; 11 Cleveland Clinic Akron General, Akron, OH USA; 12 Blue Ridge Cancer Center, Roanoke, VA USA; 13 Breast Care Specialists, Allentown, PA USA; 14 St. Clair Hospital, Pittsburgh, PA USA; 15 Texas Tech University, Lubbock, TX USA; 16 Ashikari Breast Center, Sleepy Hollow, NY USA; 17 Zucker School of Medicine, Hofstra University, Hempstead, NY USA; 18 Breast Institute, University of Oklahoma Health Sciences, Oklahoma City, OK USA; 19 Stephenson Cancer Center, Oklahoma City, OK USA; 20 Regional Breast Care, Fort Myers, FL USA; 21 Genesis Care, Fort Myers, FL USA; 22 University of Nebraska Medical Center, Omaha, NE USA; 23 The Breast Institute at JFK Medical Center, Atlantis, FL USA; 24 Baptist MD Anderson Cancer Center, Jacksonville, FL USA; 25 Exempla Saint Joseph Hospital, Denver, CO USA; 26 Vail Health, Vail, CO USA; 27 New York Medical College, Valhalla, NY USA; 28 Northwell Health Physician Partners, Mount Kisco, NY USA; 29 Phelps and Northern Westchester Hospitals, Westchester, NY USA; 30 St. Mary Medical Alliance Cancer Specialists, Langhorne, PA USA; 31 Agendia Inc., Irvine, CA USA

## Abstract

**Background:**

The Neoadjuvant Breast Symphony Trial (NBRST) demonstrated the 70-gene risk of distant recurrence signature, MammaPrint, and the 80-gene molecular subtyping signature, BluePrint, precisely determined preoperative pathological complete response (pCR) in breast cancer patients. We report 5-year follow-up results in addition to an exploratory analysis by age and menopausal status.

**Methods:**

The observational, prospective NBRST (NCT01479101) included 954 early-stage breast cancer patients aged 18–90 years who received neoadjuvant chemotherapy and had clinical and genomic data available. Chemosensitivity and 5-year distant metastasis-free survival (DMFS) and overall survival (OS) were assessed. In a post hoc subanalysis, results were stratified by age (≤ 50 vs. > 50 years) and menopausal status in patients with hormone receptor-positive/human epidermal growth factor receptor 2-negative (HR+/HER2−) tumors.

**Results:**

MammaPrint and BluePrint further classified 23% of tumors to a different subtype compared with immunohistochemistry, with more precise correspondence to pCR rates. Five-year DMFS and OS were highest in MammaPrint Low Risk, Luminal A-type and HER2-type tumors, and lowest in MammaPrint High Risk, Luminal B-type and Basal-type tumors. There was no significant difference in chemosensitivity between younger and older patients with Low-Risk (2.2% vs. 3.8%; *p* = 0.64) or High-Risk tumors (14.5% vs. 11.5%; *p* = 0.42), or within each BluePrint subtype; this was similar when stratifying by menopausal status. The 5-year outcomes were comparable by age or menopausal status for each molecular subtype.

**Conclusion:**

Intrinsic preoperative chemosensitivity and long-term outcomes were precisely determined by BluePrint and MammaPrint regardless of patient age, supporting the utility of these assays to inform treatment and surgical decisions in early-stage breast cancer.

**Supplementary Information:**

The online version contains supplementary material available at 10.1245/s10434-022-11666-2.

Neoadjuvant chemotherapy (NCT) has been used to downstage primary breast cancers in order to facilitate surgical management, and the extent of response to NCT has been shown to predict long-term outcome.^1^ Preoperative treatment decisions for patients with early-stage breast cancer have traditionally been based on clinical pathological features, including age, lymph node status, histological grade, and receptor status. These factors are limited in accurately reflecting the complete biological profile of an individual patient’s tumor and do not reliably predict chemotherapy benefit. Breast cancer management has evolved towards using multigene diagnostic signatures, which evaluate a robust number of genomic biomarkers simultaneously, thus capturing the underlying molecular mechanism(s) of tumor cell progression and metastasis.^2^ MammaPrint evaluates the expression of 70 genes to determine whether an early-stage breast cancer patient is at low risk or high risk of distant recurrence.^3–5^ The phase III, prospective randomized, MINDACT trial demonstrated the ability of MammaPrint to identify Low-Risk patients who have excellent outcomes without chemotherapy despite a clinical high-risk assessment, confirming the test’s value in guiding adjuvant chemotherapy decisions.^6^

Despite its diverse biology, breast cancer is clinically subtyped based only on hormone receptors (HRs; estrogen receptor [ER] and progesterone receptor [PR]) and human epidermal growth factor receptor 2 (HER2) status using immunohistochemistry (IHC) and fluorescence in situ hybridization (FISH); however, gene expression signatures can increase the precision by which functional pathways regulated by these receptors are defined.^7,8^ The BluePrint signature captures activated downstream molecular pathways based on the expression of 80 genes and classifies tumors into functional subtypes: Luminal-type, HER2-type, or Basal-type.^9,10^ Luminal-type tumors are further stratified into Luminal A-type or Luminal B-type based on MammaPrint Low-Risk and High-Risk results, respectively. Additionally, MammaPrint genes were selected using unsupervised hierarchical clustering to assess the metastatic potential of a tumor, whereas BluePrint genes were selected in a supervised training approach based on concordant IHC receptor status to determine the intrinsic molecular subtypes of a breast tumor.^3,9^ Although both MammaPrint and BluePrint genes capture the 10 hallmarks of cancer,^11^ only four genes overlap between the two signatures. The precision of BluePrint and MammaPrint molecular stratification has been demonstrated in retrospective studies^12–14^ and prospectively with the Neoadjuvant Breast Symphony Trial (NBRST), a multi-institutional registry study, which showed that in an interim cohort of 426 patients, BluePrint molecular subtyping further stratified 22% of tumors into a different molecular subtype compared with IHC/FISH assessment.^15–17^ The addition of BluePrint classification resulted in improved precision in determining treatment response rates before surgery, indicating that identification of molecular subgroups based on gene expression may better inform neoadjuvant treatment decisions.

In this study, we report 5-year outcomes according to MammaPrint and BluePrint stratification in 954 NCT-treated early-stage breast cancer patients enrolled in NBRST. Furthermore, three important clinical studies using two different genomic assays recently demonstrated premenopausal patients or patients aged ≤ 50 years with HR-positive/HER2-negative (HR+/HER2−), low genomic risk breast cancer exhibit a chemotherapy benefit. This observation was in contrast to postmenopausal patients or patients aged > 50 years who did not benefit from chemotherapy despite identical genomic and clinical features.^18–20^ Therefore, in a post hoc exploratory subanalysis, the relationship between age and menopausal status and the prognostic capability of MammaPrint and BluePrint in determining chemosensitivity and 5-year overall survival (OS) and distant metastasis-free survival (DMFS) was evaluated.

## Methods

### Patients

The NBRST prospectively enrolled patients from July 2011 to December 2014 across 67 institutions in the US. The protocol was approved by Institutional Review Boards at all participating sites and was registered with ClinicalTrials.gov (NCT01479101). This study was conducted in accordance with the ethical standards as established in the Declaration of Helsinki. All patients consented to study participation, clinical data collection, and publication. Patients aged 18–90 years diagnosed with histologically proven early-stage breast cancer (stage I–III) were eligible for inclusion. Additionally, patients were eligible if they started or were scheduled to start neoadjuvant systemic therapy after receiving standard-of-care MammaPrint and BluePrint testing, the results of which were made available to the treating physician. Patients were excluded if they had an excisional biopsy or axillary dissection; confirmed distant metastatic disease; tumor sample with ≤ 30% tumor cells; received any prior chemotherapy, radiotherapy, or endocrine therapy for treatment of breast cancer; and any serious uncontrolled infections or concomitant disease. Data on baseline characteristics, treatment, recurrences, and death were collected using case report forms within 6 weeks after receiving MammaPrint and BluePrint results, at 4 weeks postsurgery, at 2–3 years postsurgery, and at 5 years postsurgery. Patients with missing treatment information (*n* = 44) were not included in the final analysis. Patients received NCT or neoadjuvant endocrine therapy (NET) at the physician’s discretion adhering to either National Comprehensive Cancer Network (NCCN)-approved regimens^21^ or other established regimens.

### Molecular and Clinical Subtyping

MammaPrint and BluePrint are based on microarray gene expression analysis^6,9^ and were successfully performed on pretreatment core needle biopsies (formalin-fixed paraffin-embedded and some fresh tissue) sent to the Agendia Laboratory (Irvine, CA, USA) and blinded for clinical and pathological data. MammaPrint categorized tumors as Low Risk (MammaPrint index > 0.000) or high risk of distant recurrence (MammaPrint index ≤ 0.000). BluePrint classified tumors into Luminal-type, HER2-type, or Basal-type.^9^ MammaPrint stratified Luminal-type into Luminal A-type (Low Risk) or Luminal B-type (High Risk).^9^ HR status (ER and PR) was assessed locally by IHC and determined positive if there were ≥ 1% of tumor cells with positive nuclear staining, per American Society of Clinical Oncology/College of American Pathologists (ASCO/CAP) guidelines. HER2 was determined locally by IHC/FISH according to 2011–2014 ASCO/CAP guidelines and determined positive by 3+ staining or FISH positivity.^22,23^ IHC/FISH classified tumors as HR+/HER2−, HR+/HER2+, HR−/HER2+, or triple-negative (TN; HR−/HER2−).

### Objectives and Endpoints

The primary endpoint for patients who received NCT was pathological complete response (pCR), defined as the absence of invasive carcinoma in both breast and axilla at microscopic examination of the resected specimen at the time of surgery, regardless of the presence of carcinoma in situ (ypT0/isN0). DMFS was the primary endpoint and OS was the secondary endpoint for long-term follow-up. In a post hoc exploratory subanalysis, pCR rates, DMFS, and OS probabilities were stratified by age (≤ 50 vs. > 50 years) and menopausal status (pre- vs. post-) in patients with HR+/HER2− tumors; due to the small sample size (*n* = 2), HR+/HER2− tumors that were BluePrint HER2-type were not included in this analysis.

### Statistical Analysis

NBRST was designed as an observational, exploratory study, therefore sample size calculation was not utilized because only descriptive statistics were initially planned. Descriptive statistics were used to summarize age, race/ethnicity, menopausal status, histologic tumor type, tumor stage, grade, MammaPrint and BluePrint results, and IHC/FISH subtypes. pCR rates were reported for patients treated with NCT and were calculated for each BluePrint/MammaPrint molecular subtype and compared with rates for IHC/FISH subtypes using a two-tailed *z*-test for proportions. The same test was used to compare response rates by age group and menopausal status. Clinical characteristics were categorized by age group; Chi-square test or Fisher’s exact test were used to identify differences. Statistical significance was defined by a two-sided *p*-value of < 0.05 for all tests. The probability of pCR as a function of the MammaPrint Index was calculated.

For survival analyses, 5-year DMFS and OS survival curves were estimated using the Kaplan–Meier method, and log-rank test determined survival differences. Time to DMFS was calculated from date of diagnosis to date of first distant metastasis, death of any cause if no recurrence, or censored at the last follow-up date. Time to OS was calculated from diagnosis date to death from any cause, or censored at the last follow-up date. The association of MammaPrint and BluePrint with time to DMFS event was assessed by Cox regression models for patients with HR+/HER2− tumors, which are the most common breast cancer clinical subtype and the focus population of the post hoc exploratory subanalysis. Adjusted hazard ratio (HR) and 95% confidence interval (CI) were estimated for MammaPrint and BluePrint separately. Clinical parameters used to calculate clinical risk, including lymph node status, grade, and tumor stage, were chosen as covariates for Cox regression modeling. The proportional hazards assumption was tested using Schoenfeld residuals. Statistical analyses were conducted using Stata version 16 (StataCorp LLC, College Station, TX, USA).

## Results

### Patient Characteristics and Treatment Regimens

Between 2011 and 2014, 1091 women with breast cancer aged 18–90 years were enrolled at 67 US institutions, of whom 1025 were eligible for inclusion and had known treatment information. Patients who received NCT (*n* = 954) were included in the final analysis in which molecular classification and pCR were assessed (electronic supplementary Fig. 1). Survival outcomes at 5-years were assessed in NCT-treated patients who had follow-up data available (*n* = 841). In the overall trial population, a majority of patient demographics and tumor characteristics were similar between patients who had follow-up data compared with patients who were lost to follow-up (electronic supplementary Table 1). Statistically significant differences were observed in ethnicity, histologic type, and IHC/FISH subtype between both groups, however these differences are numerically small.

The median age was 52-years (range 18–89); 44% of patients (*n* = 421) were premenopausal and 55% (*n* = 521) were postmenopausal (Table 1). Patients self-identified as Caucasian (72%), African American (15%), Hispanic (9%), Asian (2%), or ‘other’ (1%). A majority of breast tumors were invasive ductal carcinoma (IDC; 88%), T2 or T3 (78%), and had intermediate or high histologic grade (90%). At diagnosis, 57% of patients were lymph node-positive by clinical assessment, with or without lymph node biopsy. Based on IHC/FISH, 45% of tumors were HR+/HER2−, 30% were HER2+ (19% HR+ and 11% HR−), and 25% were TNBC (Table 1). Treatment was based on IHC/FISH subtyping rather than genomic classification (electronic supplementary Tables 2 and 3). For patients receiving postoperative systemic adjuvant therapy, treatment was according to IHC/FISH subtype (electronic supplementary Tables 4 and 5).

**TABLE 1:** Clinical characteristics for patients with early-stage breast cancer who received neoadjuvant chemotherapy (*n* = 954) classified according to BluePrint and MammaPrint

Characteristics	Luminal A-type [*n* = 118]	Luminal B-type [*n* = 313]	HER2-type [*n* = 166]	Basal-type [*n* = 357]	Total [*n* = 954]
Median age, years (range)	53 (32–79)	54 (22–79)	52 (23–81)	52 (18–89)	52 (18–89)
*Race/ethnicity*					
Caucasian	99 (83.9)	221 (70.6)	118 (71.1)	253 (70.9)	691 (72.4)
African American	10 (8.5)	49 (15.7)	19 (11.4)	68 (19.0)	146 (15.3)
Hispanic	6 (5.1)	29 (9.3)	23 (13.9)	27 (7.6)	85 (8.9)
Asian	2 (1.7)	10 (3.2)	5 (3.0)	3 (0.8)	20 (2.1)
Other	1 (0.8)	4 (1.3)	1 (0.6)	6 (1.7)	12 (1.3)
*Menopausal status*					
Pre	52 (44.1)	137 (43.8)	72 (43.4)	160 (44.8)	421 (44.1)
Post	63 (53.4)	174 (55.6)	92 (55.4)	192 (53.8)	521 (54.6)
Unknown	3 (2.5)	2 (0.6)	2 (1.2)	5 (1.4)	12 (1.3)
*Histologic type*					
IDC	88 (74.6)	262 (83.7)	150 (90.4)	336 (94.1)	836 (87.6)
ILC	22 (18.6)	26 (8.3)	3 (1.8)	7 (2.0)	58 (6.1)
Mixed IDC/ILC	5 (4.2)	16 (5.1)	9 (5.4)	5 (1.4)	35 (3.7)
Other	3 (2.5)	9 (2.9)	4 (2.4)	9 (2.5)	25 (2.6)
*T stage*					
T1	14 (11.9)	40 (12.8)	25 (15.1)	59 (16.5)	138 (14.5)
T2	62 (52.5)	170 (54.3)	87 (52.4)	211 (59.1)	530 (55.6)
T3	35 (29.7)	76 (24.3)	35 (21.1)	68 (19.0)	214 (22.4)
T4	7 (5.9)	22 (7.0)	15 (9.0)	19 (5.3)	63 (6.6)
TX	0	5 (1.6)	4 (2.4)	0	9 (0.9)
*N stage*					
N0	52 (44.1)	94 (30.0)	55 (33.1)	164 (45.9)	365 (38.3)
N1	52 (44.1)	173 (55.3)	91 (54.8)	145 (40.6)	461 (48.3)
N2	7 (5.9)	24 (7.7)	10 (6.0)	23 (6.4)	64 (6.7)
N3	3 (2.5)	5 (1.6)	2 (1.2)	12 (3.4)	22 (2.3)
NX	4 (3.4)	17 (5.4)	8 (4.8)	13 (3.6)	42 (4.4)
*Grade*					
G1	43 (26.9)	20 (5.9)	2 (1.2)	5 (1.4)	70 (6.8)
G2	87 (54.4)	151 (44.7)	66 (39.3)	48 (13.4)	352 (34.3)
G3	21 (13.1)	150 (44.4)	94 (55.9)	305 (85.0)	570 (55.6)
GX	9 (5.6)	17 (5.0)	6 (3.6)	1 (0.2)	33 (3.2)
*IHC/FISH classification*^*a*^					
HR+/HER2− (luminal)	99 (83.9)^b^	240 (76.7)^c^	2 (1.2)	85 (23.9)^d^	426 (44.7)
HR+/HER2+ (HER2)	18 (15.3)	63 (20.1)	83 (50.0)	19 (5.3)	183 (19.2)
HR−/HER2+ (HER2)	0	3 (1.0)	78 (47.0)	23 (6.5)	104 (10.9)
Triple negative (basal)	1 (0.8)	7 (2.2)	3 (1.8)	229 (64.3)^e^	240 (25.2)
*MammaPrint*					
Low risk	118	0	3 (1.8)	0	121 (12.7)
High risk	0	313	163 (98.2)	357	833 (87.3)

Data are expressed as *n* (%) unless otherwise specified

For each clinical characteristic, percentages were calculated by column

*IHC* immunohistochemistry, *FISH* fluorescence in situ hybridization, *IDC* invasive ductal carcinoma, *ILC* invasive lobular carcinoma, *HR* hormone receptor, *HER2* human epidermal growth factor receptor 2

^a^Missing clinical subtype information for one patient with BluePrint Basal-type tumor

^b^Three HER2 IHC/FISH equivocal

^c^Twelve HER2 IHC/FISH equivocal

^d^Two HER2 IHC/FISH equivocal

^e^Ten HER2 IHC/FISH equivocal

### Further Classification of Immunohistochemistry and Fluorescence In Situ Hybridization (IHC/FISH) Subtypes by BluePrint and MammaPrint

**Fig. 1:**
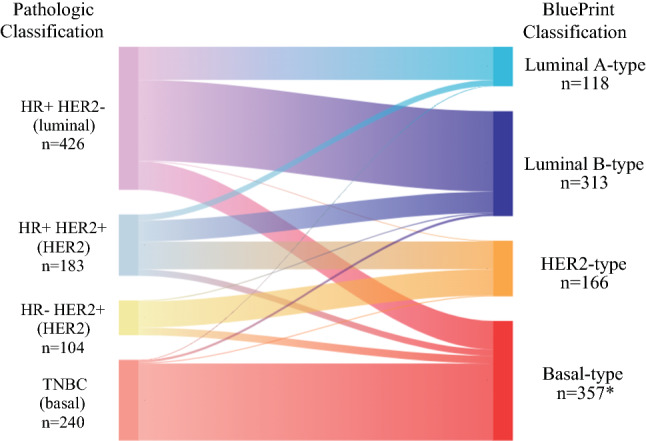
Sankey diagram depicting further stratification of IHC/FISH-defined tumors (left) by BluePrint/MammaPrint (right) in patients with early-stage breast cancer who received NCT (*n* = 954; *one patient with Basal-type tumor and missing pathologic subtype information was excluded). *IHC* immunohistochemistry, *FISH* fluorescence in situ hybridization, *NCT* neoadjuvant chemotherapy, *HR* hormone receptor, *HER2* human epidermal growth factor receptor 2, *TNBC* triple-negative breast cancer

MammaPrint classified 87% of tumors as High Risk and 13% as Low Risk (Table 1). BluePrint and MammaPrint classified 12% of tumors as Luminal A-type, 33% as Luminal B-type, 17% as HER2-type, and 37% as Basal-type. Overall, 23% of patients were further stratified into a different molecular subgroup compared with IHC/FISH (Table 1 and Fig. 1). Approximately 20% of HR+/HER2− tumors were BluePrint Basal-type (*n* = 85) or HER2-type (*n* = 2); 29% of IHC/FISH-defined HER2+ tumors were Luminal-type (*n* = 84) and 15% were Basal-type (*n* = 42). Of TNBC tumors, 3% (*n* = 8) were Luminal-type and 1.3% (*n* = 3) were HER2-type.

### Pathological Complete Response Rates to Neoadjuvant Chemotherapy Based on BluePrint and MammaPrint versus IHC/FISH Classification

**Fig. 2:**
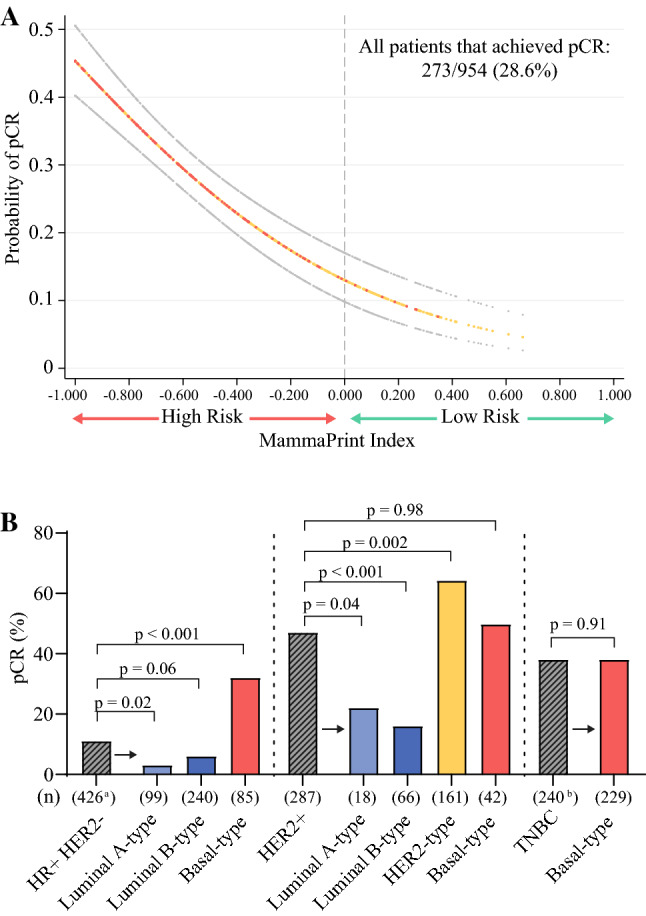
Treatment response in breast cancer patients who received NCT (*n* = 954). **A** Probability of pCR (ypT0/isN0) to NCT as a function of the MammaPrint index (*n* = 954). Red and yellow circles represent patients who did and did not have a pCR, respectively. Grey circles represent 95% confidence intervals. **B** pCR rates in IHC/FISH-defined tumors (lined bar graphs) compared with pCR rates of their respective BluePrint classifications (solid bar graphs) in NCT-treated patients (*n* = 953; 1 patient missing pathologic subtype information). Light blue represents Luminal A-type, dark blue represents Luminal B-type, orange represents HER2-type, and red represents Basal-type. Significance was assessed by a two-tailed z-test for proportions. Numbers (*n*) along the x-axis represent the total number of patients in each subgroup. ^a^Two patients with IHC-defined HR+/HER2− tumors that were classified as BluePrint HER2-type are not shown. ^b^Eleven patients with IHC-defined TNBC tumors that were classified as non-Basal type are not shown. *NCT* neoadjuvant chemotherapy, *pCR* pathological complete response, *IHC* immunohistochemistry, *FISH* fluorescence in situ hybridization, *HER2* human epidermal growth factor receptor 2, *HR* hormone receptor, *TNBC* triple-negative breast cancer

MammaPrint index was significantly associated with probability of pCR (*p* < 0.001), and a genomic High-Risk result was highly associated with pCR (Fig. 2A). Of all patients who achieved a pCR (*n* = 273), 3% (*n* = 9) had MammaPrint Low-Risk tumors and 97% (*n* = 264) had MammaPrint High-Risk tumors. Overall, BluePrint identified more tumors that achieved a pCR as genomically HER2-type and Basal-type, and more tumors that were less responsive to NCT as genomically Luminal-type (Fig. 2B). HR+/HER2− tumors that were confirmed Luminal A-type or Luminal B-type by BluePrint had a pCR rate of 3% (*n* = 3/99) and 6% (*n* = 15/240), respectively, which was lower compared with an 11% pCR in all HR+/HER2− tumors (*n* = 45/426) (Fig. 2B). In contrast, HR+/HER2− tumors that were further stratified to Basal-type had significantly higher pCR than all HR+/HER2− tumors (32% [*n* = 27/85] vs. 11% [*n* = 45/426]; *p* < 0.001). IHC/FISH-defined HER2+ tumors had a significantly higher pCR rate (47%; *n* = 136/287) than those that were further classified as Luminal A-type (22%, *n* = 4/18; *p* = 0.04) or Luminal B-type (17%, *n* = 11/66; *p* < 0.001), and a significantly lower rate than tumors confirmed as HER2-type by BluePrint (63%, *n* = 101/161; *p* = 0.002). Lastly, pCR rates were similar (*p* = 0.91) between BluePrint Basal-type tumors (38%, *n* = 88/229) and IHC/FISH-defined TNBC tumors (38%, *n* = 91/240).

### Five-Year Prognostic Stratification According to BluePrint and MammaPrint

**Fig. 3:**
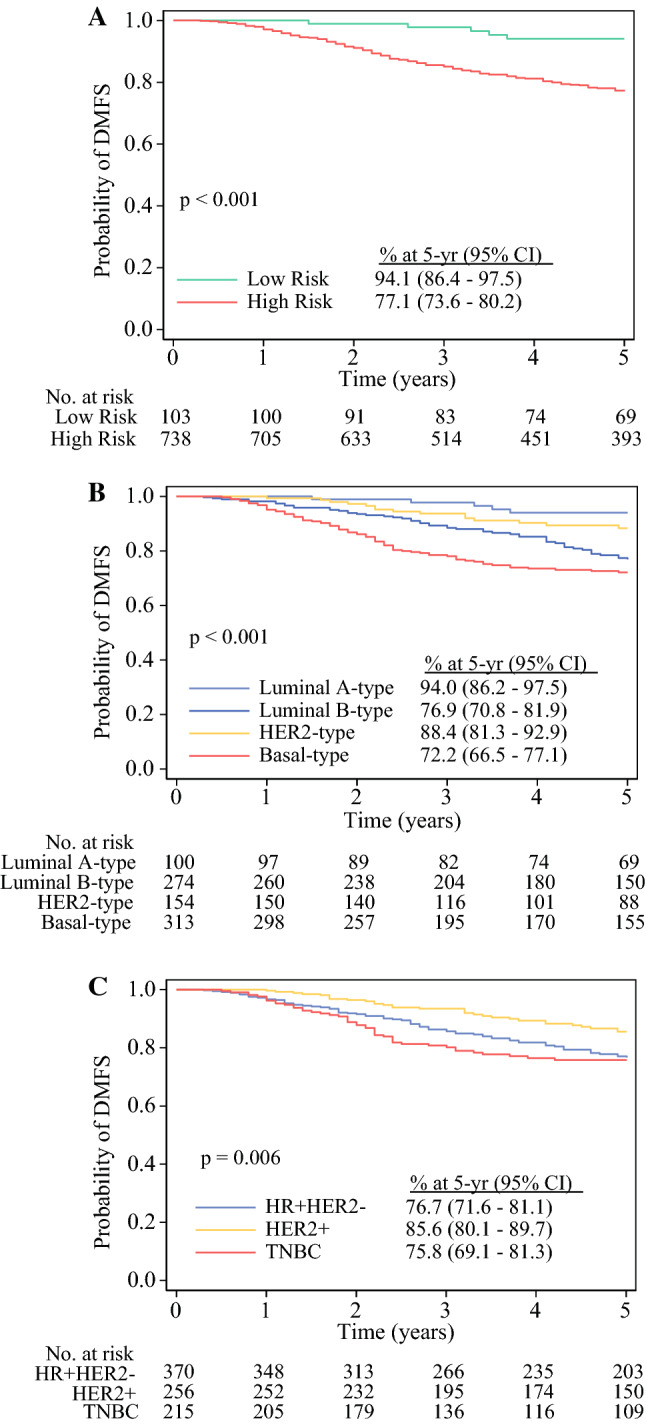
Five-year DMFS probability according to **A** MammaPrint, **B** BluePrint/MammaPrint, and **C** IHC/FISH subtyping in NCT-treated early-stage breast cancer patients with follow-up data available (*n* = 841). Significance was assessed by log-rank test. *DMFS* distant metastasis-free survival, *IHC* immunohistochemistry, *FISH* fluorescence in situ hybridization, *NCT* neoadjuvant chemotherapy, *CI* confidence interval, *HER2* human epidermal growth factor receptor, *HR* hormone receptor, *TNBC* triple-negative breast cancer

Follow-up data (median of 5.3-years) were reported for 841 patients treated with NCT, with or without HER2-targeted therapy. At 5-years, DMFS was significantly lower (*p* < 0.001) in MammaPrint High-Risk tumors (77.1%, 95% CI 73.6–80.2) compared with MammaPrint Low-Risk tumors (94.1%, 95% CI 86.4–97.5) (Fig. 3A). This result was similar for 5-year OS (electronic supplementary Fig. 2a).

The 5-year DMFS was highest in Luminal A-type (94.0%, 95% CI 86.2–97.5) and HER2-type tumors (88.4%, 95% CI 81.3–92.9), and lowest in Luminal B-type (76.9%, 95% CI 70.8–81.9) and Basal-type tumors (72.2%, 95% CI 66.5–77.1; *p* < 0.001) (Fig. 3B). This result was similar when evaluating 5-year OS (electronic supplementary Fig. 2b). Most (82.3%) DMFS events in Basal-type tumors occurred within 3 years postdiagnosis, in contrast to 40.0% and 54.7% of DMFS events in Luminal A-type and Luminal B-type tumors, respectively. Compared with IHC/FISH, MammaPrint and BluePrint could discriminate 5-year DMFS between Low-Risk and High-Risk Luminal-type tumors compared with all ER+ and/or PR+, HER2− tumors (Fig. 3B, C). BluePrint HER2-type tumors had 88.4% 5-year DMFS versus 85.6% for IHC/FISH-defined HER2+ tumors, whereas BluePrint Basal-type tumors had 72.2% 5-year DMFS versus 75.8% for TNBC tumors (Figs. 3B, C).

Multivariable Cox regression analyses were performed in NCT-treated patients with HR+/HER2− tumors to determine the association of clinical and molecular tumor characteristics with time to DMFS event (Table 2). MammaPrint High-Risk tumors had nearly a fivefold higher risk of a DMFS event than MammaPrint Low-Risk tumors, and Basal-type tumors had at least a sevenfold higher risk of a DMFS event than Luminal A-type tumors. Lymph node status, grade, and tumor stage were not significantly associated with 5-year DMFS on multivariate analysis after adjusting for molecular subtype.

**Table 2:** Multivariable analysis for DMFS in patients with HR+/HER2− tumors who received NCT

Category	HR	95% CI	*p*-value
MammaPrint			0.001*
Low risk	1 (ref)		
High risk	4.74	1.86–12.07	
Lymph node status			0.13
Negative	1 (ref)		
Positive	1.54	0.88–2.68	
Grade number			0.53
1	1 (ref)		
2	0.62	0.26–1.45	
3	0.70	0.31–1.62	
T stage			0.06
1	1 (ref)		
2	2.05	0.63–6.67	
3	3.33	1.00–11.07	
4	3.90	1.03–14.80	
BluePrint			< 0.001*
Luminal A-type	1 (ref)		
Luminal B-type	4.38	1.71–11.26	
Basal-type	7.43	2.60–21.19	
Lymph node status			0.08
Negative	1 (ref)		
Positive	1.64	0.94–2.87	
Grade number			0.48
1	1 (ref)		
2	0.63	0.29–1.49	
3	0.59	0.25–1.39	
T stage			0.05
1	1 (ref)		
2	1.91	0.59–6.24	
3	3.19	0.96–10.61	
4	4.05	1.07–15.40	

Patients with HR+/HER2− tumors who received NCT were included in the Cox regression analysis (*n* = 426)

*DMFS* distant metastasis-free survival, *HR* hormone receptor, *HER2* human epidermal growth factor receptor, *NCT* neoadjuvant chemotherapy, *HR* hazard ratio, *CI* confidence interval

^*^p-values represent statistical significance. Proportional hazards assumption is tested with *p* = 0.25 for the Cox model evaluating MammaPrint and *p* = 0.10 for the Cox model evaluating BluePrint

### Five-Year Outcome According to MammaPrint and BluePrint Stratified by Age and Menopausal Status

**Fig. 4:**
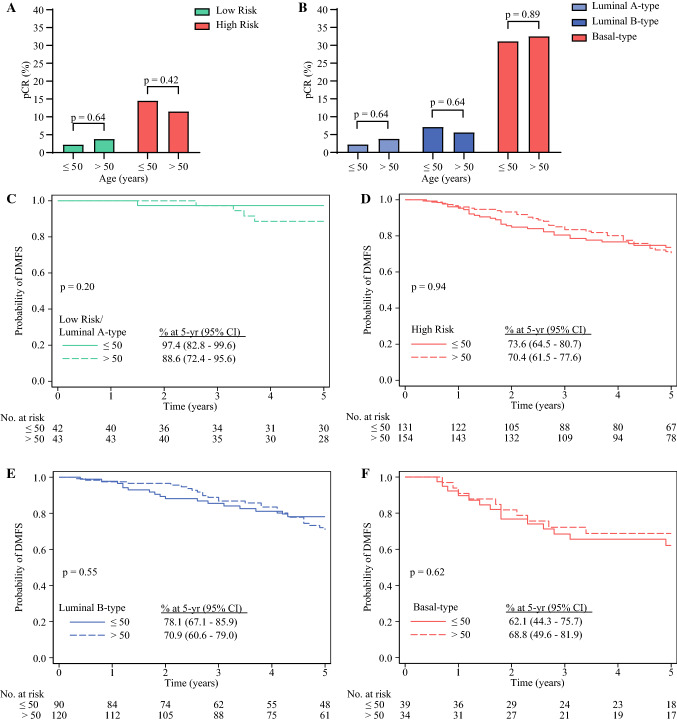
pCR rates in patients ≤ 50 years of age and patients > 50 years of age with HR+/HER2− tumors who received NCT (*n* = 426) based on their **A** MammaPrint risk and **B** BluePrint/MammaPrint classification. Significance was evaluated by a two-tailed z-test for proportions. **C**–**F** Five-year DMFS probability in patients with HR+/HER2− tumors who received NCT and had follow-up data available (*n* = 370) stratified by age: **c** MammaPrint Low-Risk, Luminal A-type tumors; **D** MammaPrint High-Risk tumors; **E** Luminal B-type tumors, and **F** Basal-type tumors. Significance was evaluated by log-rank test. *pCR* pathological complete response, *HR* hormone receptor, *HER2* human epidermal growth factor receptor, *NCT* neoadjuvant chemotherapy, *DMFS* distant metastasis-free survival, *CI* confidence interval

Among 426 patients with HR+/HER2− tumors treated with NCT, 191 were aged ≤50 years, and 235 were aged >50 years (electronic supplementary Table 6). Histologic type, tumor stage, grade, and MammaPrint risk were similar between younger and older patients. There was a higher frequency of BluePrint Basal-type tumors among younger HR+/HER2− patients. Ethnicity and lymph node status significantly differed by age group (electronic supplementary Table 6). Among MammaPrint Low-Risk patients, there was no significant difference (*p* = 0.64) in pCR rates in patients aged ≤50 years (2.2%, *n* = 1/46) compared with patients aged >50 years (3.8%, *n* = 2/53) (Fig. 4A). pCR rates were comparable between younger (14.5%, *n* = 21/145) and older (11.5%, *n* = 21/182) patients with MammaPrint High-Risk tumors (*p* = 0.42*)*. Similarly, there was no significant difference in pCR between patients aged ≤50 years versus patients aged >50 years with Luminal A-type (2.2% [*n* = 1/46] vs. 3.8% [*n* = 2/53]; *p* = 0.64), Luminal B-type (7.1% [*n* = 7/98] vs. 5.6% [*n* = 8/142]; *p* = 0.64), and Basal-type tumors (31.1% [*n* = 14/45] vs. 32.5% [*n* = 13/40]; *p* = 0.89) (Fig. 4B). Consistent with treatment response rates, there was no difference in 5-year DMFS and OS by age among MammaPrint Risk groups and BluePrint subtypes (Fig. 4C–F and electronic supplementary Figs. 2c–f).

**Fig. 5:**
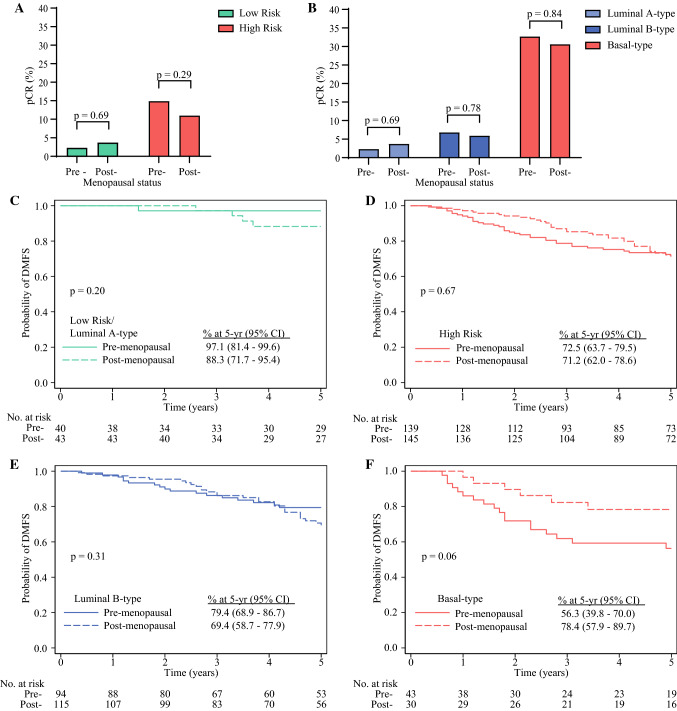
pCR rates in premenopausal and postmenopausal patients with HR+/HER2− tumors who received NCT (*n* = 423; three patients with unknown menopausal status were not included in the analysis) based on their **A** MammaPrint risk and **B** BluePrint/MammaPrint classification. Significance was evaluated by a two-tailed *z*-test for proportions. **C**–**F** Five-year DMFS probability in NCT-treated patients with HR+/HER2− tumors who had follow-up data available (*n* = 367) stratified by menopausal status: **c** MammaPrint Low-Risk, Luminal A-type tumors; **D** MammaPrint High-Risk tumors; **E** Luminal B-type tumors; and **F** Basal-type tumors. Significance was evaluated by log-rank test. *pCR* pathological complete response, *HR* hormone receptor, *HER2* human epidermal growth factor receptor, *NCT* neoadjuvant chemotherapy, *DMFS* distant metastasis-free survival, *CI* confidence interval

Of those patients with HR+/HER2− tumors treated with NCT, 197 were premenopausal (88% of whom were aged ≤ 50 years) and 226 were postmenopausal (93% of whom were aged >50 years). Of premenopausal patients, 22% (*n* = 43) were MammaPrint Low Risk and 78% (*n* = 154) were MammaPrint High Risk. Of postmenopausal patients, 24% (*n* = 54) were Low Risk and 76% (*n* = 172) were High Risk. Similar pCR rates were observed in premenopausal versus postmenopausal patients with MammaPrint Low-Risk tumors (2.3% [*n* = 1/43] vs. 3.7% [*n* = 2/54]; *p* = 0.69) or MammaPrint High-Risk tumors (14.9% [*n* = 23/154] vs. 11% [*n* = 19/172]; *p* = 0.29) (Fig. 5A). Furthermore, pCR rates were comparable between premenopausal versus postmenopausal patients according to molecular subtype (Fig. 5B). The 5-year DMFS and OS were not statistically significantly different between premenopausal and postmenopausal patients in each MammaPrint risk category and BluePrint subtype (Fig. 5C–F and electronic supplementary Fig. 3).

## Discussion

This study is the first and largest prospective trial evaluating the clinical utility of a multigene molecular subtyping signature in a preoperative setting with 5-year follow-up. Consistent with previous interim published data,^15^ MammaPrint and BluePrint further stratified 23% of tumors into a different molecular subgroup compared with IHC/FISH, translating into increased precision in determining neoadjuvant treatment response and prognostic stratification at 5-years of each molecular subtype. MammaPrint Low-Risk, Luminal A-type tumors had excellent outcomes, with a 5-year DMFS rate of 94.0% despite a low pCR rate (3%), suggesting that MammaPrint identifies patients who are unlikely to experience a survival benefit from chemotherapy and may avoid overtreatment. Longer follow-up is needed for patients with Luminal A-type tumors in this study. However, the recent updated results from the MINDACT trial demonstrated that patients with a MammaPrint Low-Risk result have excellent 9-year DMFS (89.4%, 95% CI 86.8–91.5) when treated with endocrine therapy alone, with small magnitude of benefit (2.6%) from adding chemotherapy to endocrine therapy.^20^ BluePrint detected fewer HER2-type tumors compared with IHC/FISH. However, genomic HER2-type tumors had a 14% increase in chemosensitivity and nearly 3% improvement in 5-year DMFS compared with IHC/FISH-defined HER2+ tumors.

Overall, Basal-type tumors had the worst outcome, with a 5-year DMFS and OS rate of 72.2% and 73.7%, respectively. In contrast to other molecular subtypes, most DMFS events occurred within the first 3-years postdiagnosis in Basal-type tumors. It should be noted that while recurrences will continue beyond 5-years postdiagnosis in Luminal A tumors, risk of recurrence is highest within 5-years in Luminal B tumors.^24,25^ Therefore, this finding highlights the critical need to identify patients with BluePrint Basal-type tumors who may benefit from additional systemic therapy postsurgery. There were more BluePrint Basal-type tumors than were identified within IHC/FISH-defined TN tumors due to further classification of some HR+ tumors (18%) or HER2+ tumors (14%) to Basal-type. Moreover, HR+, genomically Basal-type tumors have previously been shown to exhibit IHC ER positivity ranging from 1 to 99%.^26^ Significantly higher pCR rates for HR+ Basal-type tumors were observed, compared with HR+ Luminal-type tumors, and were similar to pCR rates observed for TN tumors. Moreover, HR+/HER2− tumors that were Basal-type had poor 5-year DMFS and OS probabilities. Lastly, MammaPrint High Risk and Basal-type classification were associated with a significantly high risk of a DMFS event, whereas grade and lymph node status were not associated with 5-year DMFS on multivariate analysis. Other molecular subtyping classifiers, such as PAM50, have demonstrated similar chemosensitivity rates but were not prognostic for DMFS in a multivariate analysis of neoadjuvant-treated patients with ER+ tumors.^27^ Together, these data strongly support that compared with clinical factors, BluePrint and MammaPrint classification is more accurate in determining response and outcome to chemotherapy. Future studies will investigate the relationship between treatment, chemosensitivity, and survival in specific subtypes with discordant IHC/FISH and genomic classification.

Recent analyses reported from the RxPONDER,^18^ TAILORx,^19^ and MINDACT^20^ trials showed that among patients with HR+/HER2−, genomic low-risk breast tumors, younger (≤ 50 years of age) or premenopausal patients benefited from chemotherapy, whereas older (> 50 years of age) or postmenopausal patients did not. Whether this difference is due to an age-dependent direct cytotoxic effect from chemotherapy or a secondary chemotherapy-induced ovarian function suppression is unknown. Indeed, younger breast cancer patients have a worse prognosis than older patients and are more likely to present with more aggressive breast cancer subtypes. However, in the current study, there was no difference in pCR to chemotherapy according to age or menopausal status, after correcting for MammaPrint risk and BluePrint subtype, suggesting no intrinsic genomic difference in chemosensitivity in breast cancers due to age or menopausal status. In line with this finding, a recent whole transcriptome analysis comparing HR+/HER2− breast cancers from patients aged ≤ 50 years versus patients aged > 50 years revealed no substantial differences in gene expression between tumors from both age groups, including Low-Risk Luminal-type tumors.^28^ Together, these data provide insight into age-related differences in chemotherapy benefit observed in these trials and suggest these differences may more likely be due to differences in host characteristics rather than age-related differences in tumor biology.^29^

Limitations of the post hoc age-based analysis include low power to detect small significant differences in response and DMFS and the inability to assess outcome in younger patients who did not receive NCT, due to small sample size. Future studies are needed to validate the findings in this report in a larger, robustly powered study. Similarly, there was no significant difference in pCR rates by menopausal status. Although not statistically significant, premenopausal patients with HR+/HER2− tumors that were classified as Basal-type had lower 5-year DMFS rates than postmenopausal patients. This observation will be the subject of future investigation. Lastly, there was a 7–12% discordance between stratifying patients by age versus menopausal status, confirming that age is not an accurate surrogate for menopausal status.

## Conclusion

MammaPrint and BluePrint testing improved the precision in determining chemosensitivity and 5-year outcomes compared with traditional pathological subtyping, supporting the clinical utility of these assays in improving neoadjuvant and subsequent surgical treatment decisions. Observed outcomes and prediction of pCR were independent of age or menopausal status, suggesting no intrinsic differences in breast cancer chemosensitivity, in addition to confirming the utility of MammaPrint and BluePrint findings regardless of age.

## Supplementary Information

Below is the link to the electronic supplementary material.

Supplementary file1 (DOCX 40664 kb)
