# Supplementary material for: Age-Independent Preoperative Chemosensitivity and 5-Year Outcome Determined by Combined 70- and 80-Gene Signature in a Prospective Trial in Early-Stage Breast Cancer
Source: Ann Surg Oncol. 2022 Apr 4;29(7):4141–52. doi: 10.1245/s10434-022-11666-2 (PMC9174138; doi:10.1245/s10434-022-11666-2)
Supplement: Supplementary file 1 — Supplementary file1 (DOCX 40664 kb) [file 10434_2022_11666_MOESM1_ESM.docx]

**Supplemental Information**

**Age-independent preoperative chemosensitivity and 5-year outcome determined by combined 70- and 80-gene signature in a prospective trial in early-stage breast cancer**

Pat Whitworth, MD, FACS, FSSO^1,2^; Peter D. Beitsch, MD, FACS^2,3^; James V. Pellicane, MD^4^; Paul L. Baron, MD^5,6^; Laura A. Lee, MD^7^; Carrie L. Dul, MD^8^; Charles H. Nash III, MD^9^; Mary K. Murray, MD, FACS^10,11^; Paul D. Richards, MD, FACP^12^; Mark Gittleman, MD^13^; Raye Budway, MD^14^; Rakhshanda Layeequr Rahman, MD^15^; Pond Kelemen, MD^16,17^; William C. Dooley, MD, FACS^18,19^; David T. Rock, MD^20,21^; Ken Cowan, MD^22^; Beth-Ann Lesnikoski, MD, FACS^23,24^; Julie L. Barone, DO^25,26^; Andrew Y. Ashikari, MD, FACS^16,27,28,29^; Beth Dupree, MD^30^; Shiyu Wang, MS^31^; Andrea R. Menicucci, PhD^31^; Erin B. Yoder, MS^31^; Christine Finn, BS^31^; Lisa E. Blumencranz, PhD^31^; William Audeh, MD^31*^; NBRST Investigators Group

***Corresponding Author**:

Dr. William Audeh M.D., M.S.

Medical Affairs, Agendia Inc.

22 Morgan Irvine, CA, 92618

[william.audeh@agendia.com](mailto:william.audeh@agendia.com)

(310) 849-0561

**Supplemental Fig. 1.** NBRST participant diagram

**
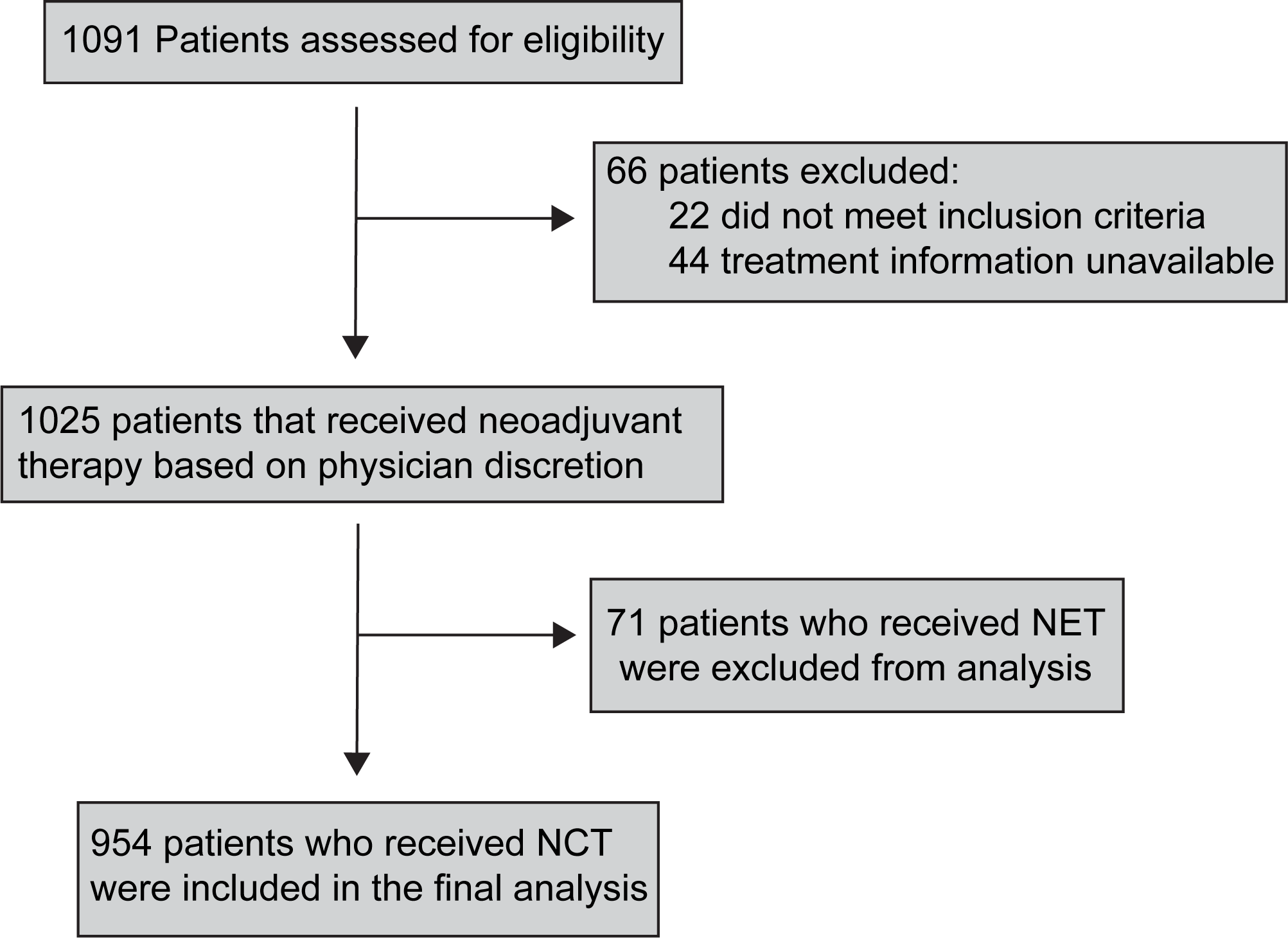
**

1091 patients were assessed for eligibility, of whom 22 were excluded because they did not meet the inclusion criteria, and 44 were excluded because treatment information was unavailable. Of 1025 eligible patients, 71 patients who received neoadjuvant endocrine therapy (NET) were excluded from the analysis. The final analysis included 954 patients who received neoadjuvant chemotherapy (NCT).

**Supplemental Table 1**. Clinical characteristics of all eligible patients enrolled in the NBRST trial (n = 1025)

|  | Patient population n = 1025 | Patients with follow-up n = 907 | Patients lost to follow-up n = 118 | P-values |
| --- | --- | --- | --- | --- |
| Median age (range), years | 53 (18-89) | 53 (18 – 89) | 56 (27 - 86) | 0.068 |
| Race/Ethnicity^a^, n (%) |  |  |  | 0.006* |
| Caucasian | 751 (73.3) | 668 (73.6) | 83 (70.3) | 0.445 |
| African American | 150 (14.6) | 122 (13.5) | 28 (23.7) | 0.003* |
| Hispanic | 88 (8.6) | 85 (9.4) | 3 (2.5) | 0.013* |
| Asian | 22 (2.1) | 20 (2.2) | 2 (1.7) | 0.719 |
| Other | 14 (1.4) | 12 (1.3) | 2 (1.7) | 0.743 |
| Menopausal status, n (%) |  |  |  | 0.167^a^ |
| Pre | 429 (41.9) | 386 (42.6) | 43 (36.4) |  |
| Post | 584 (56.9) | 509 (56.1) | 75 (63.6) |  |
| Unknown | 12 (1.2) | 12 (1.3) | 0 |  |
| Histologic Type^a^, n (%) |  |  |  | 0.005* |
| IDC | 886 (86.4) | 791 (87.2) | 95 (80.5) | 0.046* |
| ILC | 75 (7.3) | 68 (7.5) | 7 (5.9) | 0.539 |
| Mixed IDC/ILC | 36 (3.5) | 25 (2.8) | 11 (9.3) | < 0.001* |
| Other | 28 (2.7) | 23 (2.5) | 5 (4.2) | 0.286 |
| T stage, n (%) |  |  |  | 0.188 |
| T1 | 148 (14.4) | 123 (13.6) | 25 (21.2) |  |
| T2 | 574 (56.0) | 513 (56.6) | 61 (51.7) |  |
| T3 | 229 (22.4) | 207 (22.8) | 22 (18.6) |  |
| T4 | 65 (6.3) | 56 (6.2) | 9 (7.6) |  |
| TX | 9 (0.9) | 8 (0.9) | 1 (0.8) |  |
| N stage, n (%) |  |  |  | 0.320 |
| N0 | 409 (40.0) | 354 (39.0) | 55 (46.6) |  |
| N1 | 478 (46.6) | 424 (46.7) | 54 (45.8) |  |
| N2 | 64 (6.2) | 61 (6.7) | 3 (2.5) |  |
| N3 | 22 (2.1) | 20 (2.2) | 2 (1.7) |  |
| NX | 52 (5.1) | 48 (5.3) | 4 (3.4) |  |
| Grade, n (%) |  |  |  | 0.375 |
| G1 | 70 (6.8) | 61 (6.7) | 9 (7.6) |  |
| G2 | 352 (34.3) | 307 (33.8) | 45 (38.1) |  |
| G3 | 570 (55.6) | 507 (55.9) | 63 (52.9) |  |
| GX | 33 (3.22) | 32 (3.5) | 1 (0.8) |  |
| IHC/FISH classification^a^, n (%) |  |  |  | 0.026*^b^ |
| HR+HER2- (luminal) | 494 (48.2) | 433 (47.7) | 61 (51.7) | 0.419 |
| HR+HER2+ (HER2) | 186 (18.2) | 159 (17.5) | 27 (22.9) | 0.156 |
| HR-HER2+ (HER2) | 104 (10.2) | 100 (11.0) | 4 (3.4) | 0.001* |
| Triple Negative (basal) | 240 (23.4) | 215 (23.7) | 25 (21.2) | 0.543 |
| Unknown | 1 (0.1) | 0 | 1 (0.8) |  |
| MammaPrint, n (%) |  |  |  | 0.950 |
| Low Risk | 163 (15.9) | 144 (15.9) | 19 (16.1) |  |
| High Risk | 862 (84.1) | 763 (84.1) | 99 (83.9) |  |
| BluePrint, n (%) |  |  |  | 0.282 |
| Luminal A-type | 160 (15.6) | 141 (15.5) | 19 (16.1) | 0.876 |
| Luminal B-type | 338 (33.0) | 296 (32.6) | 42 (35.6) | 0.520 |
| HER2-type | 168 (16.4) | 156 (17.2) | 12 (10.2) | 0.052 |
| Basal-type | 359 (35.0) | 314 (34.6) | 45 (38.1) | 0.451 |

For each clinical characteristic, percentages were calculated by column. P-values are compared between patients with follow-up (n = 907) and patients without follow-up (n = 118). Two sample t-test was used for continuous variable, chi-square test or Fisher’s Exact test were used for categorical variables. * indicates significant p-value. ^a^For Race/Ethnicity, Histologic Type, and IHC/FISH classification: we further examine the proportion difference of between the two groups using proportional z-test. For BluePrint, proportional z-test was performed to confirm similar proportion for each subtype between two groups. ^b^unknowns were excluded from statistical analysis. IHC = Immunohistochemistry; FISH = fluorescent in situ hybridization; IDC = invasive ductal carcinoma; ILC = invasive lobular carcinoma; HR = Hormone receptor; HER2 = human epidermal growth factor receptor 2.

**Supplemental Table 2.** Neoadjuvant treatment type received in patients with early-stage breast cancer who received NCT (n = 954) based on IHC/FISH conventional subtypes

| Patients with, n (%) | HR+HER2-  n = 426 | HR+HER2+  n = 183 | HR-HER2+  n = 104 | TNBC  n = 240 | Total  n = 953^a^ |
| --- | --- | --- | --- | --- | --- |
| Any NCT | 412 (96.7) | 3 (1.6) | 2 (1.9) | 232 (96.7) | 649 (68.1) |
| Anthracycline (AC) | 16 (3.8) | 0 | 0 | 6 (2.5) | 22 (2.3) |
| Anthracycline and taxane (AC-T or TAC) | 296 (69.5) | 2 (1.1) | 2 (1.9) | 160 (66.7) | 460 (48.3) |
| Taxane (TC) | 71 (16.7) | 1 (0.5) | 0 | 23 (9.6) | 95 (10.0) |
| Anthracycline and/or taxane, and platinum agent | 13 (3.1) | 0 | 0 | 32 (13.3) | 45 (4.7) |
| Other | 16 (3.8) | 0 | 0 | 11 (4.6) | 27 (2.8) |
| Any NCT with HER2 targeted therapy | 14 (3.3)^b^ | 180 (98.4) | 102 (98.1) | 8 (3.3)^b^ | 304 (31.9) |
| AC-TH or ddAC-TH | 3 (0.7) | 32 (17.5) | 17 (16.7) | 1 (0.4) | 53 (5.6) |
| AC-THP or ddAC-THP | 1 (0.2) | 7 (3.8) | 7 (6.9) | 1 (0.4) | 16 (1.7) |
| TCH | 2 (0.5) | 70 (38.3) | 38 (37.3) | 3 (1.3) | 113 (11.9) |
| TCHP | 5 (1.2) | 48 (26.2) | 33 (32.4) | 2 (0.8) | 88 (9.2) |
| THP |  | 6 (3.3) | 3 (2.9) |  | 9 (0.9) |
| Other | 3 (0.7) | 17 (9.3) | 4 (3.9) | 1 (0.4) | 25 (2.6) |

Percentages were calculated by the number of patients in each column. HR = hormone receptor; HER2 = human epidermal growth receptor 2; TNBC = triple-negative breast cancer; NCT = neoadjuvant chemotherapy; AC = doxorubicin and cyclophosphamide; AC-T = doxorubicin and cyclophosphamide, followed by paclitaxel or docetaxel; TAC = paclitaxel or docetaxel, doxorubicin and cyclophosphamide; TC = docetaxel and cyclophosphamide; AC-TH = doxorubicin and cyclophosphamide, followed by paclitaxel and trastuzumab; AC-THP = doxorubicin and cyclophosphamide, followed by paclitaxel, trastuzumab, and pertuzumab; TCH = docetaxel or paclitaxel, carboplatin, and trastuzumab; TCHP = docetaxel or paclitaxel, carboplatin, trastuzumab, and pertuzumab; THP = docetaxel or paclitaxel, trastuzumab and pertuzumab. ^a^Missing clinical subtype information for 1 patient. ^b^Patients had tumors that exhibited low-level heterogeneity or were HER2 equivocal by IHC/FISH.

**Supplemental Table 3.** Neoadjuvant treatment type received in patients with early-stage breast cancer who received NCT (n = 954) based on BluePrint and MammaPrint molecular subtyping

| Patients with, n (%) | Luminal A-type  n = 118 | Luminal B-type  n = 313 | HER2-type  n = 166 | Basal-type  n = 357 | Total  n = 954 |
| --- | --- | --- | --- | --- | --- |
| Any NCT | 97 (82.2) | 239 (76.4) | 4 (2.4) | 310 (86.8) | 650 (68.1) |
| Anthracycline based (AC) | 3 (2.5) | 8 (2.6) | 1 (0.6) | 10 (2.8) | 22 (2.3) |
| Anthracycline and taxane based (AC-T or TAC) | 69 (58.5) | 171 (54.6) | 3 (1.8) | 217 (60.8) | 460 (48.2) |
| Taxane based (TC) | 19 (16.1) | 44 (14.1) | 0 | 32 (9.0) | 95 (10.0) |
| Anthracycline and/or taxane, and platinum agent | 3 (2.5) | 3 (1.0) | 0 | 40 (11.2) | 46 (4.8) |
| Other | 3 (2.5) | 13 (4.2) | 0 | 11 (3.1) | 27 (2.8) |
| Any NCT with HER2 targeted therapy | 21 (17.8) | 74 (23.6) | 162 (97.6) | 47 (13.2) | 304 (31.9) |
| AC-TH or ddAC-TH | 6 (5.1) | 10 (3.2) | 29 (17.5) | 8 (2.2) | 53 (5.6) |
| AC-THP or ddAC-THP | 0 | 3 (1.0) | 10 (6.0) | 3 (0.8) | 16 (1.7) |
| TCH | 7 (5.9) | 29 (9.3) | 59 (35.5) | 18 (5.0) | 113 (11.8) |
| TCHP | 6 (5.1) | 21 (6.7) | 49 (29.5) | 12 (3.4) | 88 (9.2) |
| THP | 1 (0.8) | 1 (0.3) | 6 (3.6) | 1 (0.3) | 9 (0.9) |
| Other | 1 (0.8) | 10 (3.2) | 9 (5.4) | 5 (1.4) | 25 (2.6) |

Percentages were calculated by the number of patients in each column. NCT = neoadjuvant chemotherapy; AC = doxorubicin and cyclophosphamide; AC-T = doxorubicin and cyclophosphamide, followed by paclitaxel or docetaxel; TAC = paclitaxel or docetaxel, doxorubicin and cyclophosphamide; TC = docetaxel and cyclophosphamide; AC-TH = doxorubicin and cyclophosphamide, followed by paclitaxel and trastuzumab; AC-THP = doxorubicin and cyclophosphamide, followed by paclitaxel, trastuzumab, and pertuzumab; TCH = docetaxel or paclitaxel, carboplatin, and trastuzumab; TCHP = docetaxel or paclitaxel, carboplatin, trastuzumab, and pertuzumab; THP = docetaxel or paclitaxel, trastuzumab and pertuzumab.

**Supplemental Table 4.** Adjuvant treatment received in patients that received NCT (n = 954) based on IHC/FISH subtype

| Patients with, n (%) | HR+HER2-  n = 426 | HR+HER2+  n = 183 | HR-HER2+  n = 104 | TNBC  n = 240 | Total  n = 953^a^ |
| --- | --- | --- | --- | --- | --- |
| Adjuvant Treatment^b^ | 339 (79.6) | 153 (83.6) | 87 (83.7) | 157 (65.4) | 736 (77.2) |
| CT non-trastuzumab | 43 (12.7) | 23 (15) | 12 (13.8) | 21 (13.4) | 99 (13.5) |
| CT and trastuzumab | 9 (2.7) | 74 (48.4) | 51 (58.6) | 6 (3.8) | 140 (19.0) |
| ET | 304 (89.7) | 131 (85.6) | 7 (8.0) | 12 (7.6) | 454 (61.7) |
| External RT | 265 (78.2) | 105 (68.6) | 61 (70.1) | 140 (89.2) | 571 (77.6) |
| Other | 21 (6.2) | 14 (9.2) | 3 (3.4) | 11 (7.0) | 49 (6.7) |

For each subtype, the percentage of treatment type is calculated based on the number of patients that received adjuvant treatment. HR = hormone receptor; HER2 = human epidermal growth receptor 2; TNBC = triple-negative breast cancer; CT = chemotherapy; ET = endocrine therapy; RT = radiation therapy. ^a^Missing clinical subtype information for 1 patient. ^b^Frequency of different adjuvant treatment types was not mutually exclusive.

**Supplementary Table 5.** Adjuvant treatment received in patients that received NCT (n = 954) based on molecular subtype

| Patients with, n (%) | Luminal A-type  (n = 118) | Luminal B-type  (n = 313) | HER2-type  (n = 166) | Basal-type  (n = 357) | Total  (n = 954) |
| --- | --- | --- | --- | --- | --- |
| Adjuvant Treatment^a^ | 96 (81.4) | 257 (82.1) | 141 (84.9) | 242 (67.8) | 736 (77.1) |
| CT non-trastuzumab | 7 (7.3) | 31 (12.1) | 21 (14.9) | 40 (16.5) | 99 (13.5) |
| CT and trastuzumab | 11 (11.5) | 31 (12.1) | 74 (52.5) | 24 (9.9) | 140 (19.0) |
| ET | 91 (94.8) | 232 (74.1) | 68 (48.2) | 63 (26.0) | 454 (61.7) |
| External RT | 76 (79.2) | 191 (74.3) | 101 (71.6) | 203 (56.9) | 571 (77.6) |
| Other | 6 (6.3) | 18 (7) | 9 (6.4) | 16 (6.6) | 49 (6.7) |

For each subtype, the percentage of treatment type is calculated based on the number of patients that received adjuvant treatment. CT = chemotherapy; ET = endocrine therapy; RT = radiation therapy. ^a^Frequency of different adjuvant treatment types was not mutually exclusive.

**Supplemental Figure 2.** 5-year OS according to BluePrint and MammaPrint and stratified by age

**
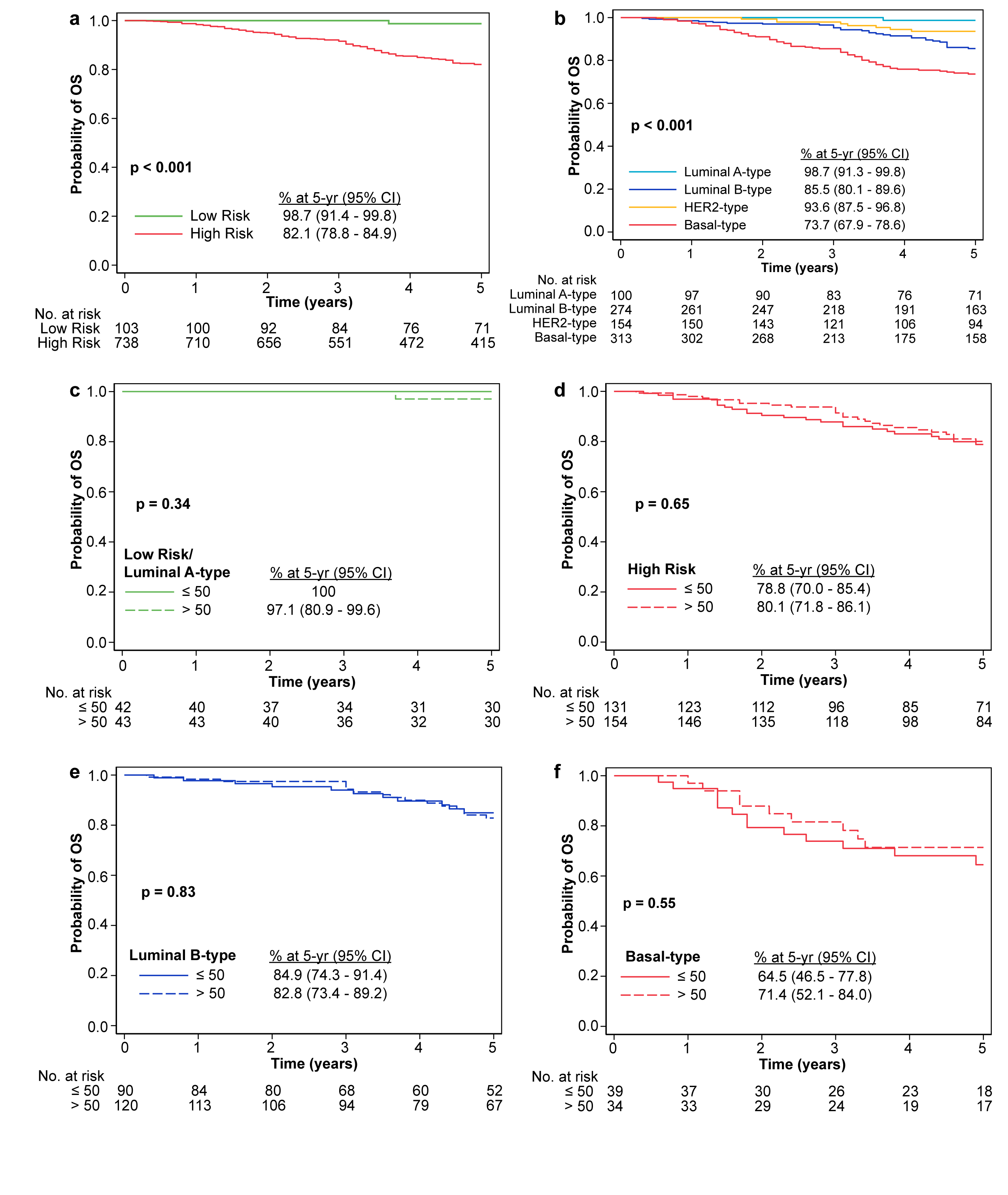
**

**(a-b) 5-year** OS probability according to MammaPrint **(a)** and BluePrint/MammaPrint **(b)** in NCT treated patients with early-stage breast cancer who had follow-up data available (n = 841). **(c-f)** 5-year OS probability in NCT treated patients with HR+HER2- tumors who had follow-up data available (n = 370) stratified by age: **(c)** MammaPrint Low Risk, Luminal A-type tumors, **(d)** MammaPrint High Risk tumors, **(e)** Luminal B-type tumors, and **(f)** Basal-type tumors. Significance was evaluated by log-rank test.

**Supplemental Table 6**. Clinical characteristics of patients aged ≤ 50 or > 50 with HR+HER2-, early-stage breast cancer who received NCT (n = 426)

| Characteristics | ≤ 50 years old  n = 191 | > 50 years old n = 235 | p-Value |
| --- | --- | --- | --- |
| Menopausal status^a^, n (%) |  |  |  |
| Pre- | 174 (92.1) | 23 (9.8) | < 0.001 |
| Post- | 15 (7.9) | 211 (90.2) |  |
| Race/Ethnicity, n (%) |  |  |  |
| Caucasian | 132 (69.1) | 186 (79.1) | 0.03 |
| African American | 28 (14.7) | 29 (12.4) |  |
| Hispanic | 21 (11.0) | 13 (5.5) |  |
| Asian | 4 (2.1) | 6 (2.6) |  |
| Other | 6 (3.1) | 1 (0.4) |  |
| Histologic Type, n (%) |  |  |  |
| IDC | 163 (85.3) | 188 (80.0) | 0.38 |
| ILC | 15 (7.9) | 30 (12.8) |  |
| Mixed IDC/ILC | 9 (4.7) | 10 (4.3) |  |
| Other | 4 (2.1) | 7 (3.0) |  |
| T stage, n (%) |  |  |  |
| T1 | 28 (14.7) | 18 (7.7) | 0.07 |
| T2 | 101 (52.9) | 133 (56.6) |  |
| T3 | 53 (27.7) | 62 (26.4) |  |
| T4 | 8 (4.2) | 20 (8.5) |  |
| TX | 1 (0.5) | 2 (0.8) |  |
| N stage, n (%) |  |  |  |
| N0 | 54 (28.3) | 84 (35.7) | 0.03 |
| N1 | 101 (52.9) | 121 (51.5) |  |
| N2 | 25 (13.1) | 12 (5.1) |  |
| N3 | 4 (2.1) | 5 (2.1) |  |
| NX | 7 (3.6) | 13 (5.6) |  |
| Grade, n (%) |  |  |  |
| G1 | 17 (8.9) | 22 (9.4) | 0.62 |
| G2 | 71 (37.2) | 99 (42.1) |  |
| G3 | 95 (49.7) | 102 (43.4) |  |
| GX | 8 (4.2) | 12 (5.1) |  |
| MammaPrint, n (%) |  |  |  |
| Low Risk | 46 (24.1) | 53 (22.6) | 0.80 |
| High Risk | 145 (75.9) | 182 (77.4) |  |
| BluePrint, n (%) |  |  |  |
| Luminal A-type | 46 (24.1) | 53 (22.6) | 0.09 |
| Luminal B-type | 98 (51.3) | 142 (60.4) |  |
| HER2-type | 2 (1.0) | 0 |  |
| Basal-type | 45 (23.6) | 40 (17.0) |  |

For each clinical characteristic, percentages were calculated by column. Chi-squared test or Fisher’s exact test were used to identify differences. Statistical significance was defined by a 2-side *p* < 0.05 for all tests. IDC = invasive ductal carcinoma; ILC = invasive lobular carcinoma; HR = hormone receptor; HER2 = human epidermal growth receptor 2. ^a^Three patients with unknown menopausal status not included.

**Supplemental Figure 3**. 5-year OS according to BluePrint and MammaPrint and stratified by menopausal status.


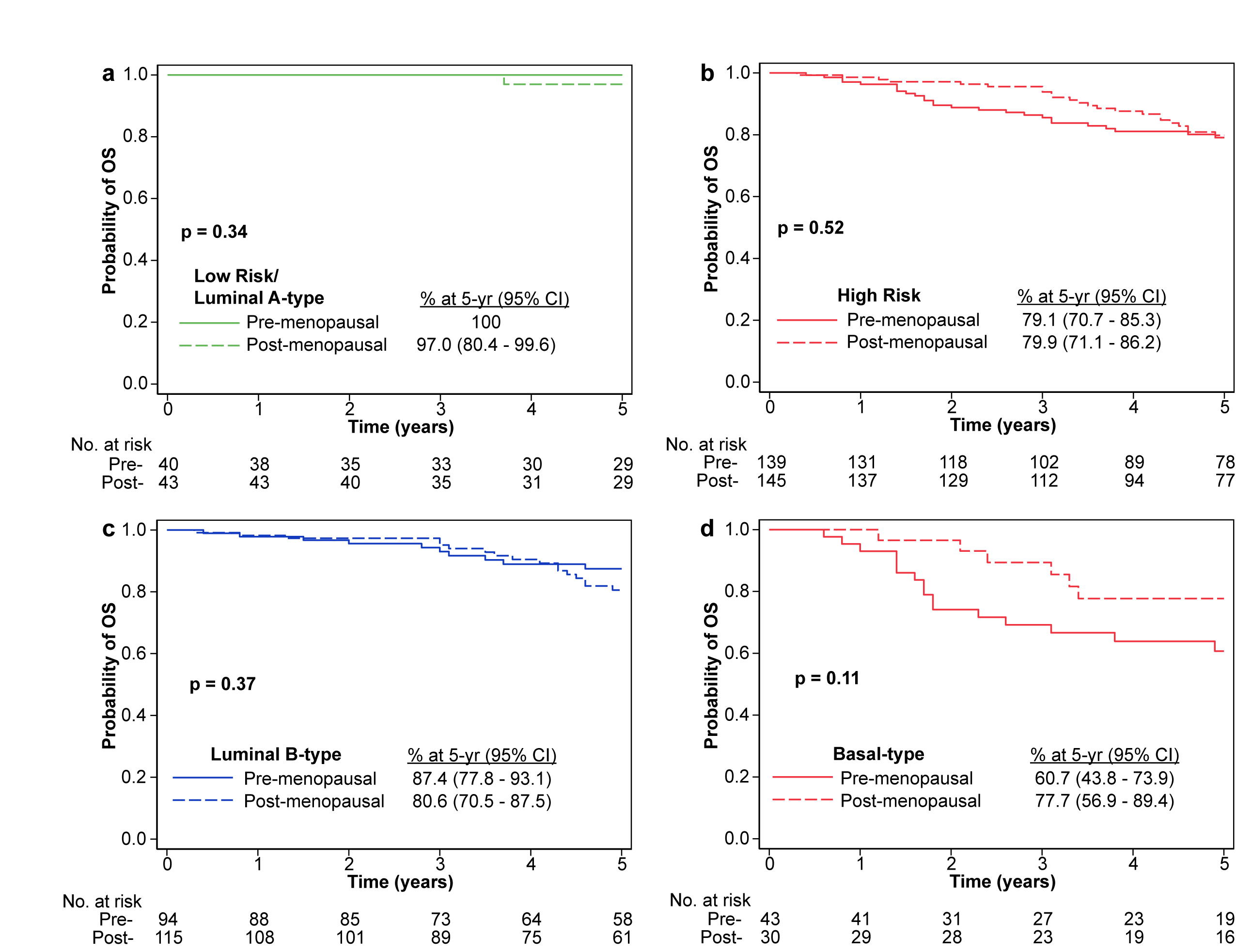


**(a-d)** 5-year OS probability in NCT treated patients with HR+HER2- tumors who had follow-up data available (n = 367; 3 patients with unknown menopausal status not included in analysis) stratified by menopausal status: **(a)** MammaPrint Low Risk, Luminal A-type tumors, **(b)** MammaPrint High Risk tumors, **(c)** Luminal B-type tumors, and **(d)** Basal-type tumors. Significance was evaluated by log-rank test.
